# Supplementary material for: Unraveling the Role of the Zinc-Dependent Metalloproteinase/HTH-Xre Toxin/Antitoxin (TA) System of Brucella abortus in the Oxidative Stress Response: Insights into the Stress Response and Virulence
Source: Toxins (Basel). 2023 Aug 31;15(9):536. doi: 10.3390/toxins15090536 (PMC10538038; doi:10.3390/toxins15090536)
Supplement: Supplementary file 1 [file toxins-15-00536-s001.zip › toxins-2489162-supplementary.pdf]

Supplementary data

>NC_007618.1:270612-271513 ("HTH-Xre transcriptional regulator" and "ImmA/IrrE family metallo-endopeptidase" of Brucella abortus 2308 chromosome I, complete sequence

ATGACCACGGAACTCGGGAAGGAGCTTAGAAAATTACGCATCGACCATAACGAACGCTTGTTGGATATGT
CCAAGAAAATTGGAAAGTCCTCGGCGTTCATCTCAGCAGTTGAAACCGGTCAAAAACCCCACCGAACGG
GTTTGAGGAACTTGTGATAGGTGCGTATCACTTGGCACGAGCTGCAGCTGAAAAGCTACGTATTGCAGCC
GATAAATCTAGGTCGGCCTTTACAATTACGGCAGACACACCCTTGAGCAGGGATACAGCAGGATTACTAG
CGAGAAAAATGAACTCGCTTTCTGATGAGCAATTGGAGGAAATCAAGCACATTCTCAGGCGAGGTAAAGA
GGAATGAGCAGTCAGAATTACGTTGTCCCACCGCTTTCGTGGGACAACATCGGCCAATTAAGCGACGCAA
TCCGCGTGCAGTTCTCTCTTGCTGATCAAGCGACATTTCCCGTCATGGATTTTCTCGAACTTGTACTGTG
CCAGCGCATGGGCATGGTCGATTTGAGGATAAAAACCCAACAAGAGATGGGGGATTTCGAGGGGTTCACT
GATCCAAAGGGCAAGTTCATTATTCTACGCGAAGACGTTTATGAGAACGCCTGCAACGATAGTCCTAGAG
ACCGGTTTACGGTAGCGCACGAATTGGGTCACTTTTTTCTTCACACTGGTATACCGATGGCGCGTGCGAG
CGACGAAAGGCGAATAAAGGACTATCGGCTGAGTGAGCCACAAGCCAATCAGTTCGCAGGAGAGCTTTTG
ATGCCTCGCCAATTCATGTCGCCGTTCGATACAGCAGAAGATGTCATGCAAAGACATAGTGTATCTCGTG
GCGCGGCAGATATCCGGCTCAATTTTATGAGAAAGAAGTGGATCAATAAAAAAGGGATCTGA

"HTH-Xre transcriptional regulator" translation to protein:
MTTELGKELRKLRIDHNERLLDMSKKIGKSSAFISAVETGQKTPPNGFEELVIGAYHLARAAAEKLRIAADKS
RSAFTITADTPLSRDTAGLLARKMNSLSDEQLEEIKHILRRGKEE"

pfam12844: HTH_19
Helix-turn-helix domain
Members of this family contains a DNA-binding helix-turn-helix domain. This family contains many example antitoxins from bacterial toxin-antitoxin systems. These antitoxins are likely to be DNA-binding domains.

"ImmA/IrrE family metallo-endopeptidase" translation to protein:
MSSQNYVVPPLSWDNIGQLSDAIRVQFSLADQATFPVMDFLELVLCQRMGMVDLRIKTQQEMGDFEGFTDPKG
KFIILREDVYENACNDSPRDRFTVAHELGHFFLHTGIPMARASDERRIKDYRLSEPQANQFAGELLMPRQFMS
PFDTAEDVMQRHSVSRGAADIRLNFMRKKWINKKGI

COG2856: ImmA: Zn-dependent peptidase ImmA, M78 family [Posttranslational modification, protein turnover, chaperones]

pfam06114: Peptidase_M78:
IrrE N-terminal-like domain: This entry includes the catalytic domain of the protein ImmA, which is a metallopeptidase containing an HEXXH zinc-binding motif from peptidase family M78. ImmA is encoded on a conjugative transposon. Conjugating bacteria are able to transfer conjugative transposons that can, for example, confer resistance to antibiotics. The transposon is integrated into the chromosome, but during conjugation excises itself and then moves to the recipient bacterium and re-integrate into its chromosome. Typically a conjugative tranposon encodes only the proteins required for this activity and the proteins that regulate it. During exponential growth, the ICEBs1 transposon of Bacillus subtilis is inactivated by the immunity repressor protein ImmR, which is encoded by the transposon and represses the genes for excision and transfer. Cleavage of ImmR relaxes repression and allows transfer of the transposon. ImmA has been shown to be essential for the cleavage of ImmR. This domain is also found in in metalloprotease IrrE, a central regulator of DNA damage repair in Deinococcaceae, HTH-type transcriptional regulators RamB and PrpC.
